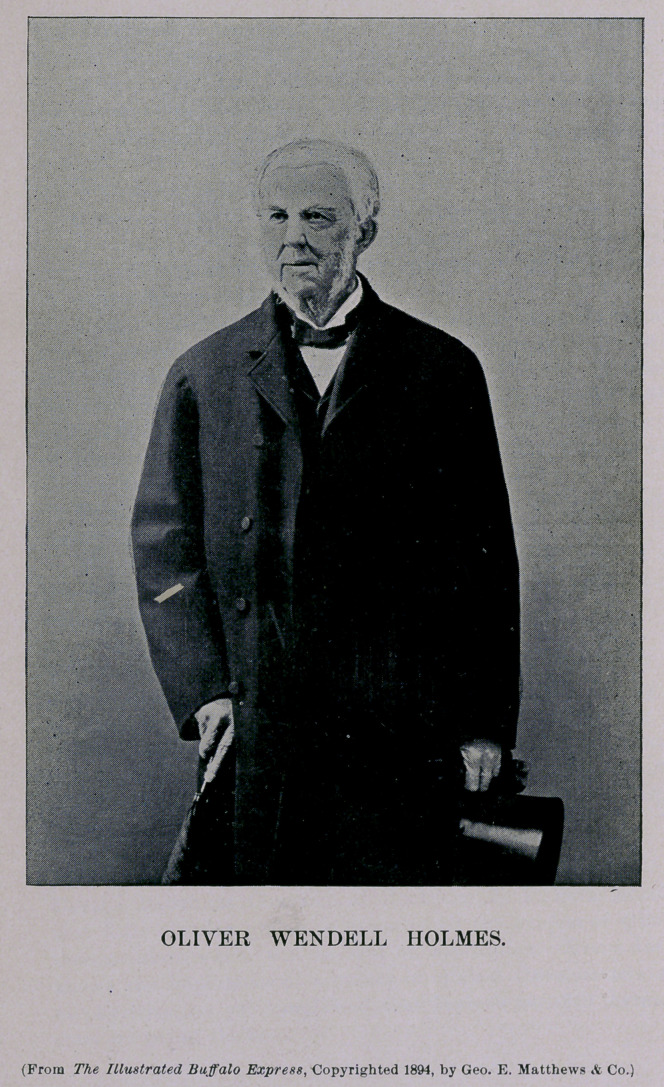# Oliver Wendell Holmes

**Published:** 1894-11

**Authors:** 


OLIVER WENDELL HOLMES.

(From *The Illustrated Buffalo Express*, Copyrighted 1894, by Geo. E. Matthews & Co.)

BUFFALO MEDICAL AND SURGICAL JOURNAL

A MONTHLY REVIEW OF MEDICINE AND SURGERY.

*EDITORS:*

THOMAS LOTHROP, M. D. - - WM. WARREN POTTER, M. D.

All communications, whether of a literary or business nature, should be addressed to the managing editor: 284 Franklin Street, Buffalo, N. Y.

Vol. XXXIV. NOVEMBER, 1894. No. 4.

### OLIVER WENDELL HOLMES.

The most distinguished American physician has passed to his immortality, and it is fitting that the Journal should take more than ordinary cognizance of an event that is and must be common to us all. Oliver Wendell Holmes died at his home at Beverly Farm, at noon, on Sunday, October 7, 1894, without pain and without warning excepting such admonition as must always come with advancing years.

Dr. Holmes's life was a remarkable one from several view points. He was born on commencement day at Cambridge, August 29, 1809, and was, therefore, at the time of his death, just past eighty-five years of age. His father was a clergyman of the Presbyterian church, and his mother, for whom his second name was given, was of Dutch descent.

He was educated first at Cambridge, then at Phillips's Academy, and finally entered Harvard College in 1825, whence he graduated four years later. He entered Harvard Law School in 1830, but soon abandoned law for medicine and took his medical degree from Harvard in 1836. Before this time he began to develop literary ability of an unusual order, and had already made a name in the poetical field through a poem before the Phi Beta Kappa Society. He also won several of the Boylston prizes offered for medical dissertations about this period of his life.

He was appointed professor of anatomy and physiology in Dartmouth College in 1838, a chair that he held for two years. He married Miss Amelia Lee Jackson, daughter of Hon. Charles Jackson, June 16, 1840, and three children were born of this marriage. Oliver Wendell Holmes, now a justice of Massachusetts

Supreme Court, the elder son, alone survives. A son and a daughter are dead.

Dr. Holmes was appointed Parkman professor of anatomy and physiology in Harvard Medical School in 1847. He taught anatomy for thirty-five years, retiring from the chair in 1882. He made his lectures attractive, no doubt largely through his literary attainments, which had now become known throughout the world as of a high order. The Autocrat of the Breakfast Table was introduced to the reading public through the columns of the *Atlantic Monthly* in 1857, and it is largely due to Dr. Holmes's contributions to that periodical that it became one of the best read magazines in the country. From the moment that he assumed the editorship of the *Atlantic*, Dr. Holmes began to be recognized throughout the world as a man of letters, and literature now claimed his attention more than medicine.

His essay, however, on Puerperal Fever, though published in an early day, long before the germ theory of disease was understood, is often quoted for its clearness and scientific reasoning and is one of the medical classics of the period. Who, too, has not been delighted with the charming literary merit of the Autocrat of the Breakfast Table and that delightful medicated novel, Elsie Venner?

While Dr. Holmes's fame in prose is world-wide, yet it is as a poet that his greatest literary merit is recognized and must endure.

Since Dr. Holmes's retirement from active duties of life, which may properly date from 1882, when he resigned his chair of anatomy at Harvard, he has been the recipient of many honors. Dinners have been given and other public ceremonies have been held commemorative of the esteem his fellow-countrymen hold him in, and he has been ever ready to respond with his pen to any reasonable demand that could be made upon him. Poems, essays, obituary notices and letters have been written without number, and he has received at his own house and entertained, in the most delightful fashion, visitors without limit, including literary men and women, savants, civilians, as well as callers from mere curiosity.

No man has contributed to the *esprit de corps* of the profession of medicine to a greater degree that has Dr. Holmes, for this certainly has been the trend even of his non-professional writings.

He leaves a legacy rich in prose and verse that will make his memory green during all the coming centuries, wherever science is known and literature is appreciated.

## Figures and Tables

**Figure f1:**